# Inclisiran: a novel drug for the treatment of dyslipidemia

**DOI:** 10.1097/MS9.0000000000000089

**Published:** 2023-01-12

**Authors:** Abdullah Nadeem, Rabeea Tariq, Areeba Basaria

**Affiliations:** Department of Medicine, Dow University of Health Sciences, Karachi, Pakistan

## Background

A significant risk factor for cardiovascular events is dyslipidemia. Increases in serum total cholesterol, low-density lipoprotein cholesterol (LDL-C), triglycerides, or a reduction in serum high-density lipoprotein cholesterol concentration are all considered signs of dyslipidemia^1^. According to the World Health Organization, dyslipidemia is thought to be a factor in over 2.6 million deaths annually^2^. Dyslipidemia appears to have a weighted prevalence that is relatively high in Pakistan. A community-based national study with 10 834 participants indicated that 96% of the participants had dyslipidemia, with the incidence of the condition being much greater in urban than rural regions^3^.

## Pathophysiology of dyslipidemia

Since it intensifies vascular changes and endothelial dysfunction, LDL-C plays a crucial role in the etiology of atherosclerotic disease. Atherosclerosis may result from the deposition of circulating lipid profile elements, notably modified LDL-C, in the tunica intima of the arterial wall^4^,^5^.

## Current treatment options for dyslipidemia

### Statins

Statins prevent the rate-limiting enzyme in the manufacture of cholesterol, 3-hydroxy-3-methylglutaryl-coenzyme A reductase. Statins have been proven to increase the chance of developing diabetes mellitus because they may interfere with insulin signaling pathways, impair the function of pancreatic beta cells, and perhaps raise insulin resistance^6^. Due to the reduction of tubular reabsorption of small molecular weight proteins, statin medication might be linked to benign proteinuria^7^.

### Ezetimibe

This cholesterol absorption inhibitor prevents the Niemann-Pick C1-like 1 protein from functioning in the digestive system (as well as in hepatocytes). LDL receptors are activated by blocking intestinal absorption of cholesterol, which lowers plasma LDL-cholesterol levels^8^.

### Fibrates

Specifically, in individuals with diabetes or metabolic syndrome, fibrates target atherogenic dyslipidemia by raising plasma high-density lipoprotein cholesterol concentrations and lowering small dense LDL particles and triglycerides (MetS)^9^.

Numerous drugs such as omega-3 fatty acid therapy, bempedoic acid, angiopoietin-like proteins, and PPARβ/δ agonists are being used for the treatment of dyslipidemias^10^.

## Inclisiran as an adjunct therapy

Inclisiran is a brand-new pharmaceutical drug that was just released on the market as an antilipemic drug to lower LDL-C levels. It works by specifically inhibiting the hepatic synthesis of the serine protease proprotein convertase subtilisin/kexin type 9 (PCSK9) in the liver, which lowers LDL-C levels^11^. The liver is largely responsible for producing and secreting PCSK9 into the bloodstream. By attaching to LDL receptors intracellularly and extracellularly and steering them toward the lysosome for destruction, PCSK9 prevents LDL receptors from recycling naturally, increasing the levels of LDL-C in the blood^12^.

In two phase III clinical studies, the ORION-10 and ORION-11 trials, a total of 1561 and 1617 individuals underwent randomization, respectively. Inclisiran, given subcutaneously every 6 months, resulted in LDL-cholesterol levels falling by about 50%^13^.

## Recommended dosage and side effects

It is recommended as a supplement to diet and the most tolerated statin medication for people with symptomatic atherosclerotic cardiovascular disease or heterozygous familial hypercholesterolemia, who need additional lowering of LDL-C. It is administered as an injectable SC solution (284 mg/1.5 ml, prefilled syringe). If necessary for treatment, measure LDL-C no later than 30 days after beginning^14^.

Local reactions, such as a minor, self-limiting rash and hyperpigmentation, musculoskeletal pain, headaches, coughing, and back pain, as well as acute nasopharyngitis or hiccups, were among the most frequently reported negative effects in studies with inclisiran 15,16.

## Conclusion and future prospect

Dyslipidemias continue to be a key contributor to atherosclerotic cardiovascular disease. It is a significant factor in mortality and morbidity in low and middle income countries. The long-term reported negative effects of traditional pharmacological therapy, notably statin medication, make it less reliable. The addition of the medication to the dyslipidemias’ current therapeutic options will provide new therapeutic opportunities. Inclisrian will lessen the number of deaths, especially in Pakistan, where cardiovascular diseases continue to be a leading cause of death among elderly patients.

## Ethical approval

Not applicable.

## Sources of funding

None.

## Authors’ contribution

A.N. contributed to conceptualization, writing – original draft, review, and editing. R.T. contributed in writing – original draft. A.B. contributed in writing – original draft.

## Conflicts of interest disclosure

The authors declare that they have no financial conflict of interest with regard to the content of this report.

## Research registration Unique Identifying number (UIN)

None.

## Guarantor

Abdullah Nadeem Department of Medicine, Dow University of Health Sciences, Karachi, Pakistan

## Consent

None applicable.
